# In Silico Analysis of Coding/Noncoding SNPs of Human *RETN* Gene and Characterization of Their Impact on Resistin Stability and Structure

**DOI:** 10.1155/2019/4951627

**Published:** 2019-05-20

**Authors:** Lamiae Elkhattabi, Imane Morjane, Hicham Charoute, Soumaya Amghar, Hind Bouafi, Zouhair Elkarhat, Rachid Saile, Hassan Rouba, Abdelhamid Barakat

**Affiliations:** ^1^Laboratoire de Génomique et Génétique Humaine, Institut Pasteur du Maroc, Casablanca, Morocco; ^2^Laboratoire de Biologie et Santé, Faculté des Sciences Ben M'Sik, Université Hassan II de Casablanca, Morocco

## Abstract

Resistin (*RETN*) is a gene coding for proinflammatory adipokine called resistin secreted by macrophages in humans. Single nucleotide polymorphisms (SNPs) in *RETN* are linked to obesity and insulin resistance in various populations. Using dbSNP, 78 nonsynonymous SNPs (nsSNPs) were retrieved and tested on a PredictSNP 1.0 megaserver. Among these, 15 nsSNPs were predicted as highly deleterious and thus subjected to further analyses, such as conservation, posttranscriptional modifications, and stability. The 3D structure of human resistin was generated by homology modeling using Swiss model. Root-mean-square deviation (RMSD), hydrogen bonds (h-bonds), and interactions were estimated. Furthermore, UTRscan served to identify UTR functional SNPs. Among the 15 most deleterious nsSNPs, 13 were predicted to be highly conserved including variants in posttranslational modification sites. Stability analysis predicted 9 nsSNPs (I32S, C51Y, G58E, G58R, C78S, G79C, W98C, C103G, and C104Y) which can decrease protein stability with at least three out of the four algorithms used in this study. These nsSNPs were chosen for structural analysis. Both variants C51Y and C104Y showed the highest RMS deviations (1.137 Å and 1.308 Å, respectively) which were confirmed by the important decrease in total h-bonds. The analysis of hydrophobic and hydrophilic interactions showed important differences between the native protein and the 9 mutants, particularly I32S, G79C, and C104Y. Six SNPs in the 3′UTR (rs920569876, rs74176247, rs1447199134, rs943234785, rs76346269, and rs78048640) were predicted to be implicated in polyadenylation signal. This study revealed 9 highly deleterious SNPs located in the human *RETN* gene coding region and 6 SNPs within the 3′UTR that may alter the protein structure. Interestingly, these SNPs are worth to be analyzed in functional studies to further elucidate their effect on metabolic phenotype occurrence.

## 1. Introduction

Genomic variation understanding is one of the major challenges of current genomics research field, due to the enormous number of genetic variations in the human genome. Single nucleotide polymorphisms (SNPs) represent the most abundant genetic variations throughout the human genome ranging between 3 and 5 million in each individual [1]. Mostly, SNPs are neutral, but some of them contribute to disease predisposition by modifying protein function or as genetic markers in order to find nearby disease-causing mutations through genetic association studies and family-based studies [2]. Scientists believe that these variants may also influence the response to some drugs [3].

SNPs that change the encoded amino acids are called nonsynonymous single nucleotide polymorphisms (nsSNPs). Nonsynonymous SNPs, forming about half of all genetic changes related to human diseases, can influence resulting protein structure and/or function with either neutral or deleterious effects [4, 5].

Moreover, the study of noncoding DNA is also important because it contains the majority of reported SNPs in human genome. Polymorphisms in 5′ and 3′ untranslated regions (UTRs) are of major interest because they can affect gene expression and posttranscriptional and posttranslational activities and thus be of functional relevance [6, 7].

Resistin is a proinflammatory adipokine which belongs to the cysteine-rich C-terminal domain proteins called resistin-like molecules (RELMs) and mainly secreted by adipocytes in rodents and macrophages in humans [8, 9]. The gene encoding resistin (*RETN*) is located on chromosome 19p13.2. It was shown that resistin is linked to several inflammatory disorders including obesity, type 2 diabetes, cardiovascular disease, and asthma [10–13]. This protein has effects which antagonize insulin action. Some studies have shown that resistin affects glucose transport and causes insulin-stimulated insulin receptor substrate-1 (IRS-1) degradation leading to insulin resistance induction [14–16]. Circulating resistin levels were reported to be significantly increased in both genetically and diet-induced obese mice and decreased with the administration of the antidiabetic drug Rosiglitazone [8].

Moreover, a case-control study on type 1 diabetes mellitus patients showed that the combination of insulin and Rosiglitazone decreased resistin and leptin levels significantly [17]. Genetic variants in *RETN* showed a significant association with circulating resistin levels. Beckers et al. identified the first missense mutation C78S in resistin in a morbidly obese proband and his obese mother. This finding encourages the study of variants in the *RETN* gene coding region to elucidate their involvement in pathogenesis [18]. It was estimated that genetic factors can explain up to 70% of the variation in circulating resistin levels [19]. However, analyses of the association between SNPs of the *RETN* gene and anthropometric variables and alterations related to obesity revealed inconsistent results [10, 20–23].

Basing on the importance of *RETN* gene in multiple inflammatory diseases, particularly metabolic abnormalities, we conducted a computational analysis using nsSNP effect predictors like SIFT, PolyPhen, PANTHER, PhD-SNP and PredictSNP. Most deleterious nsSNPs were further analyzed by conservation and stability tools. Finally, a structural analysis was conducted in order to identify the most functionally deleterious SNPs in coding and untranslated regions.

## 2. Material and Methods

### 2.1. Dataset Collection

The SNP information of *RETN* gene was collected from dbSNP (http://www.ncbi.nlm.nih.gov/snp/). The amino acid sequence of the protein (NCBI accession: NP_001180303) was retrieved from the NCBI protein database (http://www.ncbi.nlm.nih.gov/protein). The theoretical structure of resistin (PDB ID: 1LV6) was abandoned since it was not in agreement with the crystal structure available for mouse resistin now.

### 2.2. Prediction of Deleterious nsSNPs

PredictSNP1.0 (http://loschmidt.chemi.muni.cz/predictsnp1/) [24] was used as the predictor of the SNP effect on protein function. This resource is a consensus classifier that enables access to the nine best performing prediction tools: SIFT, PolyPhen-1, PolyPhen-2, MAPP, PhD-SNP, SNAP, PANTHER, PredictSNP, and nsSNPAnalyzer.

SIFT (Sorting Intolerant from Tolerant) predicts whether an amino acid substitution affects the protein function based on sequence homology and the physical properties of amino acids [25]. SIFT takes a query sequence and uses multiple alignment information to predict tolerated and deleterious substitutions in every position of the query sequence. PolyPhen-1 uses expert set of empirical rules to predict possible impact of amino acid substitutions, while PolyPhen-2 (Polymorphism Phenotyping v2) predicts the potential effect of an amino acid substitution on the structure and function of a human protein using multiple sequence alignment and structural information. MAPP (Multivariate Analysis of Protein Polymorphism) analyzes the physicochemical variation present in each column of a protein sequence alignment and predicts the impact of amino acid substitutions on the protein function [26]. PhD-SNP (Predictor of human Deleterious Single Nucleotide Polymorphisms) is a support vector machine- (SVM-) based predictor used to classify nsSNPs into human genetic disease-causing or benign mutations [27]. SNAP (screening for nonacceptable polymorphisms) is a neural network-based method used to predict functional effects of nonsynonymous SNPs using in silico derived protein information [28]. PANTHER (Protein Analysis Through Evolutionary Relationships) estimates the likelihood of a particular nsSNP to cause a functional effect on the protein using position-specific evolutionary preservation [29]. nsSNPAnalyzer uses a machine learning method called random forest to predict whether the nsSNP has a phenotypic effect [30] based on multiple sequence alignment and 3D structure information. Finally, PredictSNP1.0 displays the confidence scores generated by each tool and a consensus prediction as percentages by using their observed accuracy values to simplify comparisons [24].

### 2.3. Sequence Conservation

A ConSurf web server (http://consurf.tau.ac.il/) was used to analyze amino acid sequence conservation. This web-based algorithm predicts the crucial functional regions of a protein by estimating the degree of amino acid conservation based on multiple sequence alignment. The grade range from 1 to 9 estimates the extent of conservation of the amino acid throughout evolution. Therefore, grade 9 represents the most highly conserved residue, and the numbers descend to 1 representing the least conserved region. This tool analyzes the conservation at the nucleotide and amino acid levels.

### 2.4. Prediction of Posttranslational Modification Sites

A ModPred web server (http://www.modpred.org/) was used to predict posttranslational modification (PTM) sites; the server consists of a set of bootstrapped logistic regression models for each type of PTM, retrieved from 126,036 nonredundant PTM sites verified experimentally, the literature, and from the databases [31]. Results are given as residue, modification, score, confidence, and remarks. In this study, only medium and high confidence PTMs were taken into consideration.

### 2.5. Prediction of Change in Protein Stability

The change in protein stability due to nsSNPs was predicted using I-Mutant2.0 (http://folding.biofold.org/cgi-bin/i-mutant2.0), which is a support vector machine (SVM) web-based tool used for the automatic prediction of changes in protein stability due to SNP. It provides the predicted free energy change value (DDG) and the sign of the prediction as increase or decrease. DDG value is calculated from the unfolding Gibbs free energy value of the mutated protein minus the unfolding Gibbs free energy value of the wild type in kcal/mol. DDG > 0 means that the protein stability increased, and DDG < 0 means that the protein stability decreased [32].

The stability was also checked by a MUpro tool (http://mupro.proteomics.ics.uci.edu/). This server is based on two machine learning methods: support vector machines and neural networks. Both of them were trained on a large mutation dataset and showed accuracy above 84%.

This protein calculates a score between -1 and 1 as the confidence of prediction. The confidence score < 0 indicates that the mutation decreases the protein stability, while a confidence score > 0 means that the mutation increases the protein stability [33].

### 2.6. Scanning of UTR SNPs in the UTR Site

The 5′ and 3′ untranslated regions (UTRs) have crucial roles in degradation, translation, and localization of mRNAs as well as the regulation of protein-protein interaction. We used the UTRscan web server http://itbtools.ba.itb.cnr.it/utrscan to predict the functional SNPs in the 5′ and 3′UTRs. The UTRscan tool allows the enquirer to search user-submitted sequences for any of the motifs present in UTRsite. UTRsite derives data from UTRdb, a curated database that updates UTR datasets through primary data mining and experimental validation [7, 34]. To perform this analysis, the primary FASTA format data was submitted and the results were showed in the form of signal names and their positions in the transcript.

### 2.7. Structural Analysis

#### 2.7.1. Modeling of Native and Mutant Structure

**Figure 1:**
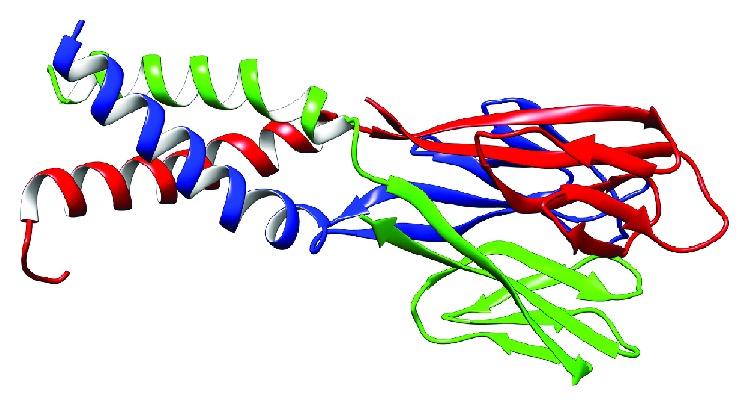
The tridimensional model of human resistin generated using homology modeling by a Swiss model web server. The model is a trimer with three chains A (red), B (green), and C (blue).

The transcript with the reference sequence NP_001180303.1 was used for the homology modeling. We selected the X-ray crystal structure of Mus musculus resistin from the Protein Data Bank (PDB) with PDB code 1RGX [9] as a template to generate a human resistin by homology modeling using the Swiss model platform (https://swissmodel.expasy.org). The model has a QMEAN of -1.83 and a sequence identity of 55.56% (Figure 1).

UCSF Chimera was used to confirm the corresponding positions of the SNPs and to construct the 15 mutant models [35]. It is a highly extensible program developed by the Resource for Biocomputing, Visualization, and Informatics at the University of California, San Francisco, for interactive visualization and analysis of molecular structures and related data.

The energy minimization of the wild type and mutant structures was performed by NOMAD-Ref server Gromacs-based as a default force field; we used conjugate gradient method for the 3D structure optimization [36].

#### 2.7.2. RMSD and Total Hydrogen Bond Prediction

UCSF Chimera served again to check RMS deviation by superimposing both native and mutant structures. In addition, this tool served to calculate total h-bond values for each structure.

#### 2.7.3. Interaction Analysis

COCOMAPS (bioCOmplexes COntact MAPS) is a web application to effectively analyze and visualize the interface in biological protein-protein complexes by making use of intermolecular contact maps. The input file was the resistin homology model in PDB format. In our study, we used COCOMAPS to analyze the interaction between the three monomers of resistin protein [37]. To achieve this, we uploaded the PDB file of resistin trimer (A, B, and C as chain IDs for each monomer) and we then compared the interaction interfaces between the two chains A and B considered as Molecule 1 interacting with the third chain C considered as Molecule 2 (interactions include residues from chain A and from chain B together interacting with chain C).

#### 2.7.4. Prediction of Protein-Protein Interactions

STRING (Search Tool for the Retrieval of Interacting Genes/Proteins, available at http://string-db.org) is a database of known and predicted protein interactions, which currently covers 9,643,763 proteins from 2031 organisms. This database provides a critical assessment and integration of protein-protein interactions including direct (physical) and indirect (functional) associations [38].

## 3. Results

### 3.1. SNP Datasets

The *RETN* SNP data investigated in this work was retrieved in early October 2018 from dbSNP database (http://www.ncbi.nlm.nih.gov/snp/?term=RETN). It contained a total of 1075 SNPs. Out of which, 78 were nsSNPs, 35 were coding synonymous SNPs, 339 were located in the noncoding region, which comprises 18 SNPs in the 5′UTR, 35 SNPs were in the 3′UTR, and 287 were in the intronic region.

### 3.2. Prediction of Deleterious nsSNPs

A total of 78 nsSNPs were selected for our investigation. This SNP collection was analyzed with various in silico prediction tools to measure their effects on pathogenicity and to find out disease-associated SNPs. All nsSNPs which were obtained from SNP database were loaded to PredictSNP1.0, and all available integrated tools were selected for prediction. Fifteen nsSNPs were predicted as deleterious by all integrated tools, except for nsSNPAnalyzer and PANTHER that did not give any prediction for any mutation. According to SNAP, a total of 38 nsSNPs out of 54 were predicted to be deleterious (70.37%), followed by MAPP with 37 deleterious nsSNPs (68.51%), PolyPhen-2 with 31 nsSNPs (57.40%), PolyPhen-1 with 25 nsSNPs (46.29%), SIFT with 26 nsSNPs (48.15%), and PhD-SNP with 18 nsSNPs (33.33%). The nsSNPs predicted as deleterious are listed in Table 1 with the expected accuracy and are selected for further analysis (Table 1).

### 3.3. Analysis of Conservation

The results of ConSurf analysis showed that 13 deleterious missense SNPs are located in highly conserved regions, with conservation values ranging between 7 and 9, which suggests that these positions are important for the resistin integrity. Among these, three residues were predicted to be exposed and functional, five others were predicted to be buried and structural, two buried residues and one exposed residue." while we should mention at the beginning of the paragraph that "11 deleterious missence SNPs are located in highly conserved regions", because we mentionned just after this that conservation values are ranging between 7 and 9 so we excluded G71 ( score: 4) and R84 (score: 6). The position 84 was predicted as moderately conserved, and the position 71 was predicted as variable residue; therefore, they were not selected for structural analysis.

### 3.4. Prediction of Posttranslational Modification Sites

ModPred was used to predict posttranslational modification sites present within the human resistin protein. Only PTMs with high or medium confidence were discussed. In the native protein, position R84 was predicted as a site of ADP-ribosylation, W98 as a site of C-linked glycosylation or proteolytic cleavage, and C103 and C104 as disulfide linkage sites. After mutagenesis, C51 appeared as a site of amidation with the change of Cys to Tyr, while the position W98 changed to a disulfide linkage site with the change of Trp to Cys. Regarding the position C104, it was predicted that the change of Cys to Tyr conferred an amidation site with a high confidence. The results of ModPred are shown in Table 2.

### 3.5. The Impact of Predicted Deleterious Mutations on Resistin Protein Stability

We analyzed the 13 missense substitutions predicted as deleterious from the previous steps with the I-Mutant2.0. and MUpro web server. nsSNPs predicted to decrease stability with both tools were selected for further structural analysis. The results are showed in Table 3.

### 3.6. Structural Analysis

#### 3.6.1. Modeling of Human Resistin Structure

**Figure 2:**
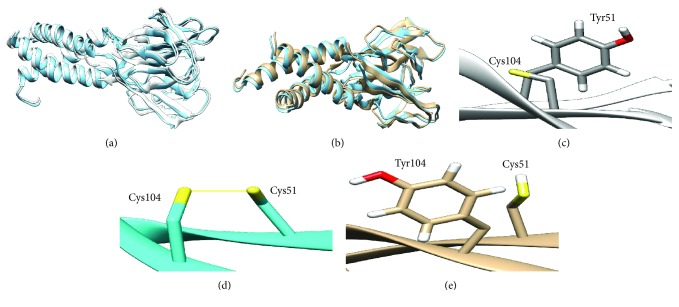
(a) Superimposed of native structure of human resistin (cyan color) onto mutant C51Y structure (white color) showing a deviation RMSD of 1.137 Å. (b) Superimposed of native structure of human resistin (cyan color) onto mutant C104Y structure (white color) showing a deviation RMSD of 1.308 Å. (c) Disulfide bond breakage in C51Y resistin mutant. (d) Disulfide bond between both cysteine residues 51 and 104 in wild-type resistin. (e) Disulfide bond breakage in C104Y resistin mutant.

Using the X-ray crystal structure (1rgx) as a template, we modeled the 3D structure of native human resistin using the Swiss model web server.  Figure 2 showed the generated model as a trimer with three monomers (A, B, and C). This trimer was used to construct the 9 mutant models of human resistin.

#### 3.6.2. RMSD Difference and Total Hydrogen Bonds

The RMSD values associated with the 9 mutants are given in Table 4. As the RMSD value increases, the deviation between native- and mutant-type structures will be higher and thus may induce a change in protein activity. Altered C51Y and C104Y mutants showed the highest RMSD; results are shown in Figures 2(a) and 2(b). In addition, total h-bonds were calculated to assess their contribution in the stability and the folding of the native protein. All mutated structures revealed a change in total h-bonds compared to the native resistin, but the C104Y mutant showed a remarkable decrease forming 254 h-bonds while the native structure formed 291. Moreover, the visualization of native structure showed that C51 and C104 residues form a disulfide bond with each other (Figure 2(d)); the change of cysteine carried on the alpha helix in these positions induces the breakage of the disulfide bridge (Figures 2(c) and 2(e)) which may disturb the protein structure.

#### 3.6.3. Interaction Analysis

The interface contacts between the amino acids present within the resistin trimer were studied using COCOMAPS. Variation in the number of different types of interactions was observed between the native and 9 resistin mutants; the results are given in Table 5.

Regarding the number of hydrophilic-hydrophilic interactions, the native complex participated with 262 hydrophilic-hydrophilic interactions. The mutant complexes I32S, C51Y, G79C, and C104Y, showed a significant increase in the number of hydrophilic-hydrophilic interactions with 286, 266, 277, and 266 interactions, respectively, which indicate a reduction in the hydrophobicity of these mutant trimers. In addition, the mutant complex C103G showed a significant increase in the number of hydrophobic-hydrophobic interactions indicating the increase of its hydrophobicity.

Moreover, we found that the C51Y mutant trimer interacts with only 75 residues of chain C forming the trimer complex while in the native complex, chain C interacts with 78 residues. This small deviation may disrupt resistin trimer formation.

#### 3.6.4. Prediction of the Effect of SNPs Located in the UTR by a UTRscan Server

The UTRscan server was used to predict the effect of UTR SNPs on transcriptional motif. Six SNPs in the 3′UTR, namely, rs920569876, rs74176247, rs1447199134, rs943234785, rs76346269, and rs78048640, were predicted to be in polyadenylation sites and thus may be responsible for pathological phenotypes. Results are given in Table 6.

**Figure 3:**
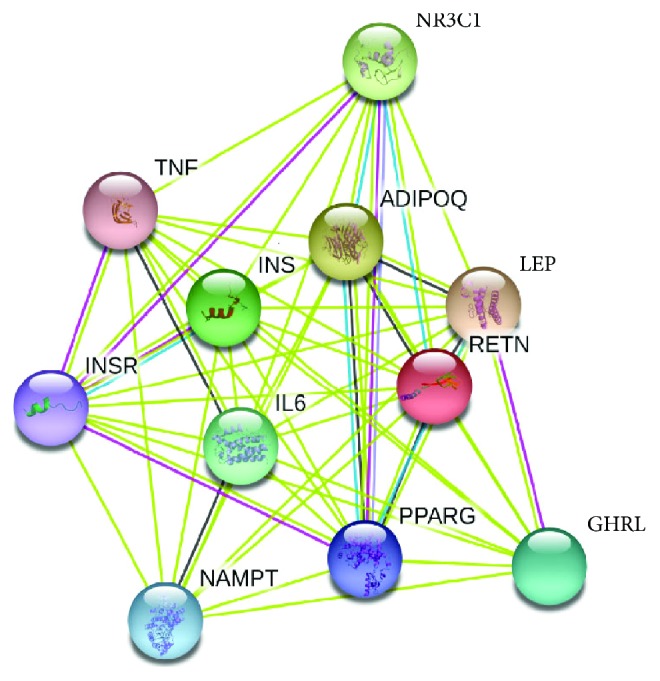
Protein-protein interaction network of resistin using a STRING server.

*Protein-Protein Interactions Using STRING*. Prediction of protein-protein interactions indicated that resistin interacts with 10 proteins including insulin (INS), insulin receptor (INSR), leptin (LEP), adiponectin (ADIPOQ), peroxisome proliferator-activated receptor gamma (PPARG), tumor necrosis factor (TNF), interleukin 6 (TNF), nicotinamide phosphoribosyltransferase (NAMPT), nuclear receptor subfamily 3 group C member 1 (NR3C1), and ghrelin/obestatin prepropeptide (GHRL) (Figure 3).

## 4. Discussion

With the growth of SNP number in databases, it becomes difficult to determine SNPs contributing in disease development. Thus, computational analysis can help to select a limited number of prioritizing deleterious SNPs for genetic disease screening [39]. Among the mutations affecting the protein function, nsSNPs are very frequently occurring in many inherited diseases [40]. Nonsynonymous SNPs were essentially described to inhibit protein activity, DNA-protein, or DNA-miRNA binding. There were many metabolic abnormalities related genes studied with computational approaches in order to predict functional SNPs such as hepatocyte nuclear factor 1 alpha (HNF1A), apolipoprotein AI (APOAI), apolipoprotein E3 (APOE3), and high-density lipoprotein (HDL) [39, 41–44]. To date, more than 70 missense mutations have been reported in human *RETN* gene. However, population-based association studies on *RETN* remain insufficient. Therefore, the current study is aimed at investigating the structural and functional impact of the SNPs present in the *RETN* gene using a thorough computational analysis. A total of 78 nsSNPs in *RETN* gene were retrieved from dbSNP database.

The methods used in this study revealed the importance of using various algorithms with different prediction capacities to estimate the effect of variations on structural and functional levels. Using a single bioinformatic tool to predict potentially pathogenic nsSNPs may not be significant [45]. Hence, the present study was based on multiple computational tools including SIFT, Poly-Phen1/2, MAAP, PhD-SNP, PredictSNP, SNAP, ConSurf, ModPred, I-Mutant2.0, and MUpro in order to identify the most deleterious nsSNPs in *RETN* gene.

Fifteen nsSNPs were predicted to be the most deleterious SNPs by these tools. In addition, using the ConSurf web server, 13 of them were predicted to be highly conserved with conservation scores ranging between 7 and 9. Moreover, PTM site prediction showed that W98 is a site for ADP-ribosylation and proteolytic cleavage in the wild type, while after mutation (W98C), it became a site for disulfide linkage with the gain of Cys residue; this may induce conformational restrictions on the protein, altering strongly its folding, function, and stability. Inversely, C103 and C104 residues were predicted as a PTM sites that lost disulfide linkages. Although disulfide bond 3D structure and interactions are highly conserved in nature, their loss may affect the protein folding. Nine nsSNPs were significantly predicted to affect stability. These results suggested that I32S, C51Y, G58E, G58R, C78S, G79C, W98C, C103G, and C104Y may be the structurally and functionally most significant SNPs in human resistin. The variant C104Y possessed the highest RMSD value, i.e., 1.308 Å followed by C51Y with an RMSD of 1.137 Å. Moreover, these variants had a significant loss of hydrogen bonds compared to the wild type. Interestingly, C51, C78, C103, and C104 residues are located in the highly conserved cysteine-rich C-terminus of resistin as all proteins of the resistin-like family share a common C-terminus domain with invariant spacing between cysteine positions (1C-X11-2C-X8-3C-X-4C-X3-5C-X10-6C-X-7C-X-8C-X9-9C-10C) [8, 46, 47]. Figures 2(c) and 2(e) showed C51Y and C104Y mutants that lost the disulfide bridge after a change of cysteine to tyrosine residues. This could be expected to destabilize the helix carrying residues that may be involved in binding sites with other proteins. Using STRING, the functional network of resistin interactions with ten different proteins implicated unanimously in inflammatory and metabolic pathways suggests the strong implication of resistin in metabolic abnormalities.

## 5. Conclusion

*RETN* gene was investigated in this work by assessing the impact of deleterious SNPs in coding and untranslated regions through a computational approach. In a total of 78 missense SNPs, 15 were predicted as the most deleterious using PredictSNP. From which, 9 nsSNPs were predicted as highly conserved and affect protein stability. The structural analysis revealed high RMSD scores for both C104Y and C51Y variants, respectively, with a loss of the total hydrogen bonds. Six UTR SNPs were predicted to be in polyadenylation sites. Hence, we concluded that 9 nsSNPs I32S, C51Y, G58E, G58R, C78S, G79C, W98C, C103G, and C104Y and 6 substitutions in the 3′UTR, namely, rs920569876, rs74176247, rs1447199134, rs943234785, rs76346269, and rs78048640, could be important candidates in the pathological process of resistin particularly in metabolic pathways.

## Figures and Tables

**Table 1:** The expected accuracy results of the SNPs of *RETN* predicted as deleterious in PredictSNP and integrated tools.

nsSNPs	ID variants	PredictSNP	PolyPhen-1	PolyPhen-2	SIFT	MAPP	PhD-SNP	SNAP
I32S	rs894321927	(0.86)	(0.74)	(0.60)	(0.79)	(0.77)	(0.85)	(0.72)
C51Y	rs759129635	(0.86)	(0.74)	(0.81)	(0.79)	(0.77)	(0.81)	(0.86)
G58R	rs760260537	(0.86)	(0.74)	(0.81)	(0.79)	(0.87)	(0.73)	(0.88)
G58E	rs763916942	(0.86)	(0.74)	(0.81)	(0.79)	(0.91)	(0.58)	(0.88)
G66R	rs566843624	(0.86)	(0.74)	(0.56)	(0.42)	(0.62)	(0.81)	(0.86)
G71R	rs772946179	(0.86)	(0.74)	(0.59)	(0.79)	(0.87)	(0.57)	(0.86)
C74W	rs532089804	(0.86)	(0.74)	(0.81)	(0.79)	(0.91)	(0.77)	(0.75)
C78S	rs199834487	(0.86)	(0.74)	(0.81)	(0.79)	(0.75)	(0.67)	(0.86)
G79C	rs111331676	(0.86)	(0.74)	(0.81)	(0.79)	(0.85)	(0.73)	(0.86)
R84C	rs779335092	(0.86)	(0.74)	(0.81)	(0.79)	(0.85)	(0.73)	(0.84)
D97E	rs768223197	(0.86)	(0.59)	(0.81)	(0.79)	(0.77)	(0.58)	(0.84)
W98C	rs776285077	(0.86)	(0.74)	(0.81)	(0.79)	(0.85)	(0.81)	(0.86)
W98L	rs1035187378	(0.86)	(0.74)	(0.81)	(0.52)	(0.91)	(0.58)	(0.88)
C103G	rs991039386	(0.86)	(0.74)	(0.81)	(0.79)	(0.76)	(0.58)	(0.88)
C104Y	rs891939673	(0.86)	(0.74)	(0.81)	(0.79)	(0.77)	(0.81)	(0.88)

**Table 2:** Details of nsSNPs selected as deleterious among the reported SNPs, their conservation analysis by ConSurf, and posttranslational modification site prediction by ModPred.

Position	Conservation score	B/E	F/S	PTM sites (wild type)	Variants	PTM sites (mutants)
I32	7	B	—	—	I32S	—
C51	9	B	S	—	C51Y	Amidation Phosphorylation
G58	9	E	F	Proteolytic cleavage	G58R	Proteolytic cleavage
					G58E	Proteolytic cleavage
G66	7	E	—	Proteolytic cleavage	G66R	Proteolytic cleavage
G71	4	B	—	—	G71R	Proteolytic cleavage ADP-ribosylation
C74	9	B	S	Disulfide linkage	C74W	—
C78	9	B	S	Disulfide linkage	C78S	O-linked glycosylation
G79	9	E	F	—	G79C	Disulfide linkage
R84	6	E	—	ADP-ribosylation Proteolytic cleavage	R84C	Disulfide linkage
D97	9	E	F	—	D97E	Disulfide linkage
W98	8	B	—	C-linked glycosylation Proteolytic cleavage Amidation	W98C	Disulfide linkage
					W98L	—
C103	9	B	S	Disulfide linkage	C103	Proteolytic cleavage
C104	9	B	S	Disulfide linkage	C104	Amidation Proteolytic cleavage

PTM: posttranslational modification; B: buried; E: exposed; F: functional; S: structural.

**Table 3:** Prediction of change in protein stability using I-Mutant2.0 and MUpro.

Position	I-Mutant2.0	MUpro		
	DDG value (kcal/mol)	DDG value	SVM	NN (kcal/mol)
I32S	**-2.45 (decrease)**	**-1.490 (decrease)**	**-0.80 (decrease)**	**-0.96 (decrease)**
C51Y	**-0.89 (decrease)**	**-0.234 (decrease)**	**-0.20 (decrease)**	**-0.69 (decrease)**
G58R	**-0.15 (decrease)**	**-0.95 (decrease)**	**-0.22 (decrease)**	**-0.73 (decrease)**
G58E	**-1.43 (decrease)**	**-0.913 (decrease)**	0.23 (increase)	**-0.67 (decrease)**
G66R	**-1.74 (decrease)**	**-0.84 (decrease)**	0.48 (increase)	0.51 (increase)
C74W	**-0.76 (decrease)**	**-0.69 (decrease)**	0.54 (increase)	0.85 (increase)
C78S	**-0.63 (decrease)**	**-1.513 (decrease)**	**-0.09 (decrease)**	0.87 (increase)
G79C	**-0.89 (decrease)**	**-0.842 (decrease)**	**-0.84 (decrease)**	**-0.38 (decrease)**
D97E	0.52 (increase)	**-0.77 (decrease)**	0.025 (increase)	**-0.65 (decrease)**
W98C	**-1.14 (decrease)**	**-0.442 (decrease)**	**-0.77 (decrease)**	**-0.92 (decrease)**
W98L	**-0.59 (decrease)**	0.16 (increase)	**-0.10 (decrease)**	0.691 (increase)
C103G	**-1.05 (decrease)**	**-1.63 (decrease)**	**-0.80 (decrease)**	0.547 (increase)
C104Y	0.32 (increase)	**-1.03 (decrease)**	**-0.36 (decrease)**	**-0.80 (decrease)**

DDG: delta delta G; SVM: support vector machine; NN: neural network.

**Table 4:** RMSD value and total hydrogen bonds after minimization of each model.

	RMSD (Å)	Total h-bonds
Native	0	291
I32S	0.023	297
C51Y	1.137	272
G58E	0.30	297
C78S	0.37	299
G58R	0.182	292
G79C	0.265	296
W98C	0.158	296
C103G	0.486	250
C104Y	1.308	254

RMSD: root-mean-square deviation.

**Table 5:** Interactions observed between the three monomeric of resistin protein in native and 9 mutant complexes.

	Native	I32S	C51Y	G58E	G58R	C78S	G79C	W98C	C103G	C104Y
Number of interacting residues Molecule 1(chain A and chain B)	129	129	130	127	129	129	128	130	130	129
Number of interacting residues Molecule 2 (chain C)	78	78	75	77	78	78	78	78	78	75
Number of hydrophilic-hydrophobic interaction	343	357	329	342	338	334	323	343	357	320
Number of hydrophilic-hydrophilic interaction	262	286	266	257	258	261	277	251	259	266
Number of hydrophobic-hydrophobic interaction	193	175	188	194	196	190	179	194	201	189

**Table 6:** SNPs (UTR mRNA) that were predicted to be functionally significant by UTRscan.

SNP ID	Nucleotide change	UTR position	Functional element change
rs920569876	A/G	3′UTR	Polyadenylation signal
rs74176247	A/G	3′UTR	Polyadenylation signal
rs1447199134	A/T	3′UTR	Polyadenylation signal
rs943234785	A/G	3′UTR	Polyadenylation signal
rs76346269	A/T	3′UTR	Polyadenylation signal
rs78048640	A/G	3′UTR	Polyadenylation signal

## Data Availability

All data generated or analysed during this study are included in this published article.
